# Through a gender lens: a scoping review of gendered experiences of AMR causes, burden and workforce in Nigeria

**DOI:** 10.3389/fgwh.2025.1523901

**Published:** 2025-05-22

**Authors:** Ayodele Oluwakemi Majekodunmi, Mabel Kamweli Aworh, Esteller Mbadiwe, Kikiope Oluwafikemi Oluwarore, Mwapu Dika Ndahi, Dooshima Kwange

**Affiliations:** ^1^ Food and Agriculture Organisation of the United Nations, Abuja, Nigeria; ^2^ Department of Biological and Forensic Sciences, Fayetteville State University, Fayetteville, NC, United States; ^3^ Ducit Blue Solutions, Abuja, Nigeria; ^4^ One Health Development Initiative, Abuja, Nigeria; ^5^ Department of Veterinary and Pest Control Services, Federal Ministry of Agriculture and Food Security, Abuja, Nigeria; ^6^ Tesedona Foundation for Animal Health, Abuja, Nigeria

**Keywords:** antimicrobial resistance (AMR), gender, inequity, infectious diseases, Nigeria

## Abstract

**Background:**

Nigeria is among the countries with the top 10 highest burdens of infectious and zoonotic diseases worldwide. There is a correspondingly high rate of antimicrobial use and misuse in humans and animals, leading to antimicrobial resistance (AMR). Antimicrobial Resistance has a very high impact on women and girls as they form the majority of health workers at community level as well as being the main care givers and livestock custodians in the home, most likely to prescribe, purchase or administer antibiotics. However, there is very little information about gendered aspects of AMR in Nigeria. This paper undertakes a scoping review of antimicrobial resistance in Nigeria through a gender lens, looking at how sex and gender interact with antimicrobial resistance and efforts to mitigate its negative effects.

**Methods:**

A PRISMA scoping review was conducted for peer-reviewed articles published from the year 2000, describing studies in Nigeria on AMR, infectious disease treatment (including treatment seeking behaviour) and access and experiences of healthcare, which either take an explicit gender approach or include sex/gender as a key variable.

**Results:**

Studies show clear gender differences in levels of disease risk/resistance, health-seeking behaviour and patterns of access to healthcare (including antimicrobials). Despite the fact that these patterns are clearly recognised across multiple publications in different settings, we did not find evidence of a corresponding analysis of how gender might reinforce these vulnerabilities.

**Conclusions:**

Gendered aspects of infectious diseases, antimicrobial access and resistance are documented in Nigeria, albeit often incidentally. This data should be taken into account when considering the AMR problem and in the design of various interventions and the design of various interventions towards improving AMR and One Health in Nigeria.

## Introduction

Antimicrobial resistance is a global health issue with significant health, financial and societal impacts, including increased morbidity, mortality, healthcare costs and productivity losses. Over 750,000 deaths annually occur as a consequence of drug-resistant bacteria and if no action is taken, this could increase to 10 million by 2050, with∼4.1 million deaths from Africa alone (1). Nigeria is among the countries with the top 10 highest burdens of infectious and zoonotic diseases worldwide (2). There is a correspondingly high rate of antimicrobial use and misuse in humans and animals, leading to antimicrobial resistance (AMR) (3, 4).

Several factors contribute to AMR in LMICs including easy access to antimicrobials without prescriptions (both medical and veterinary), low health care worker (HCW) to population ratios, and over-prescription of antimicrobials by professionals, routine use as livestock growth promoters, and poor waste management are some of the drivers of AMR in LMICs (5–7).

High levels of antimicrobial-resistant infections are recorded in humans, especially in sepsis, respiratory, and diarrheal infections (3, 8). High levels of AMR, including several patterns of MDR have also been recorded in food animal species and the environment. High levels of AM residues have also been found in food products, especially in poultry, eggs and fish (9). Resistance to over 20 commonly used disinfectants and antiseptics have also been isolated from humans, food animals and the environment (9–12). Several antimicrobials banned for use in animals in Nigeria (chloramphenicol, nitrofuran) or on the WHO or OIE critical lists (Colistin, Vancomycin, second & third generation fluoroquinolones) are routinely used in food producing animals in Nigeria (9, 13–16).

Antimicrobials are the bedrock of modern medicine. They are also vital for livestock production and husbandry. Thus, AMR threatens food production at a global level and creates enhanced potential for zoonotic disease emergence or re-emergence. People working in healthcare, agriculture and food production will also become more vulnerable, as they are increasingly exposed to resistant bacteria at work. The effects of AMR are far reaching, a threat not just to Sustainable Development Goals 2 (no hunger) and 3 (good health), but also to SDGs 1 (no poverty) 5 (gender equality) and 10 (reduced inequalities). LMICs are more susceptible to infection and are increasingly exposed to antibiotic resistant bacteria. Thus, “antibiotic resistance can breed poverty, while poverty feeds the problem of antibiotic resistance” (17).

A significant barrier to action is that AMR affects the most vulnerable people, who suffer greater impacts on their health and food systems, in addition to other development issues. Since AMR has the potential to increase existing inequalities, including gender inequality, meaningful efforts to combat AMR should take these into account.

Agriculture in Nigeria is dominated by smallholder farmers and contributes 21% to GDP, 36% to employment and 60% to non-oil export value (18). A large proportion of smallholder livestock farmers are women and girls. They are the primary care givers in homes and health facilities, also responsible for food purchase and preparation. Thus, they are affected by or fuel AMR through contact with sick people and animals, self-prescribing and dispensing of AMs, making decisions on observance of withdrawals periods and use of animal products as well as economic losses from untreatable infections.

In Nigeria, gender plays a significant role in antimicrobial resistance (AMR), particularly due to women's responsibilities in healthcare, agriculture, and informal medicine distribution. Many women, especially in rural areas, self-prescribe antibiotics for family members due to limited healthcare access while traditional birth attendants routinely administer antibiotics during childbirth without proper dosage control, contributing to resistance (19, 20). Female smallholder farmers, who dominate poultry and aquaculture, frequently misuse antibiotics to prevent livestock diseases, leading to high antimicrobial residues in food products (9). Economic losses from resistant infections disproportionately impact these women, as they rely on livestock for household income. Additionally, cultural barriers limit women's access to formal healthcare, pushing them to rely on unregulated patent medicine vendors, many of whom often women themselves sell antibiotics without prescriptions (21, 22). As primary caregivers, women also bear the burden of treating resistant infections in their families, increasing their unpaid labour and economic strain (23). Despite their critical roles, women remain underrepresented in AMR policymaking and research.

A wealth of literature examines the relationships between gender, power relations, health and infectious disease. It shows strong links between gender, poverty and women's poorer health outcomes, clearly identifying gender as one of many social determinants of health. In Nigeria, gender plays a key role in disease risk, levels and patterns of antimicrobial access, use and resistance, access to healthcare and health seeking behaviour.

Despite its position as a high priority global health issue, there is a paucity of literature that explicitly addresses sex, gender and antimicrobial resistance. In 2018, the WHO recognized the need for a focus on gender and equity issues in national AMR strategies to “understand and acknowledge how men, women and different groups in society” were differently exposed to the risk of, or affected by, antibiotic resistance” (24). This gap in evidence is mirrored in Nigeria, thus the need for a scoping review to systematically map the research done in this area and identify any existing gaps in knowledge. The specific research question is: What is known from the literature about how sex and gender interact with antimicrobial resistance to produce different experiences and perspectives of AMR.

This paper is divided into two sections. The first reviews the evidence for gendered experiences of AMR in Nigeria while the second highlights the contributions of women and women-led initiatives to mitigating AMR in Nigeria.

## Methods

This scoping review was conducted according to the PRISMA extension for scoping reviews guideline (25). To gain perspectives on gender within the AMR research, prevention and control space, expert consultations amongst female STEM professionals working in AMR across public, private and not-profit sectors were conducted.

## Eligibility criteria

Papers included in the review were selected according to the following criteria: those with a focus on or assessment of gender differences in any aspect of AMR: levels and patterns of awareness; antimicrobial access, use and stewardship; infection prevention and control.

Peer-reviewed journal papers were included if they were: published during or after 2000, written in English, based in Nigeria, described studies on AMR, infectious disease treatment (including treatment seeking behaviour) and access and experiences of healthcare, which either take an explicit gender approach or include sex/gender as a key variable. Quantitative, qualitative and mixed-method studies were included in order to consider different aspects of gender in AMR. As this is a scoping review, an inclusive approach has been used, considering all types of peer-reviewed articles as eligible, to ensure that all available data on this topic was captured.

Papers were excluded if they did not fit into the conceptual framework of the study, were based outside Nigeria, did not include sex/gender as a key variable or found no significant differences by gender.

## Search strategy

To identify potentially relevant documents, the following bibliographic databases were searched from 2000 to date: PUBMED, Web of Science, and African Journals Online (AJOL) The search strategies were drafted and further refined by initial results.

## Data screening

**Figure 1:**
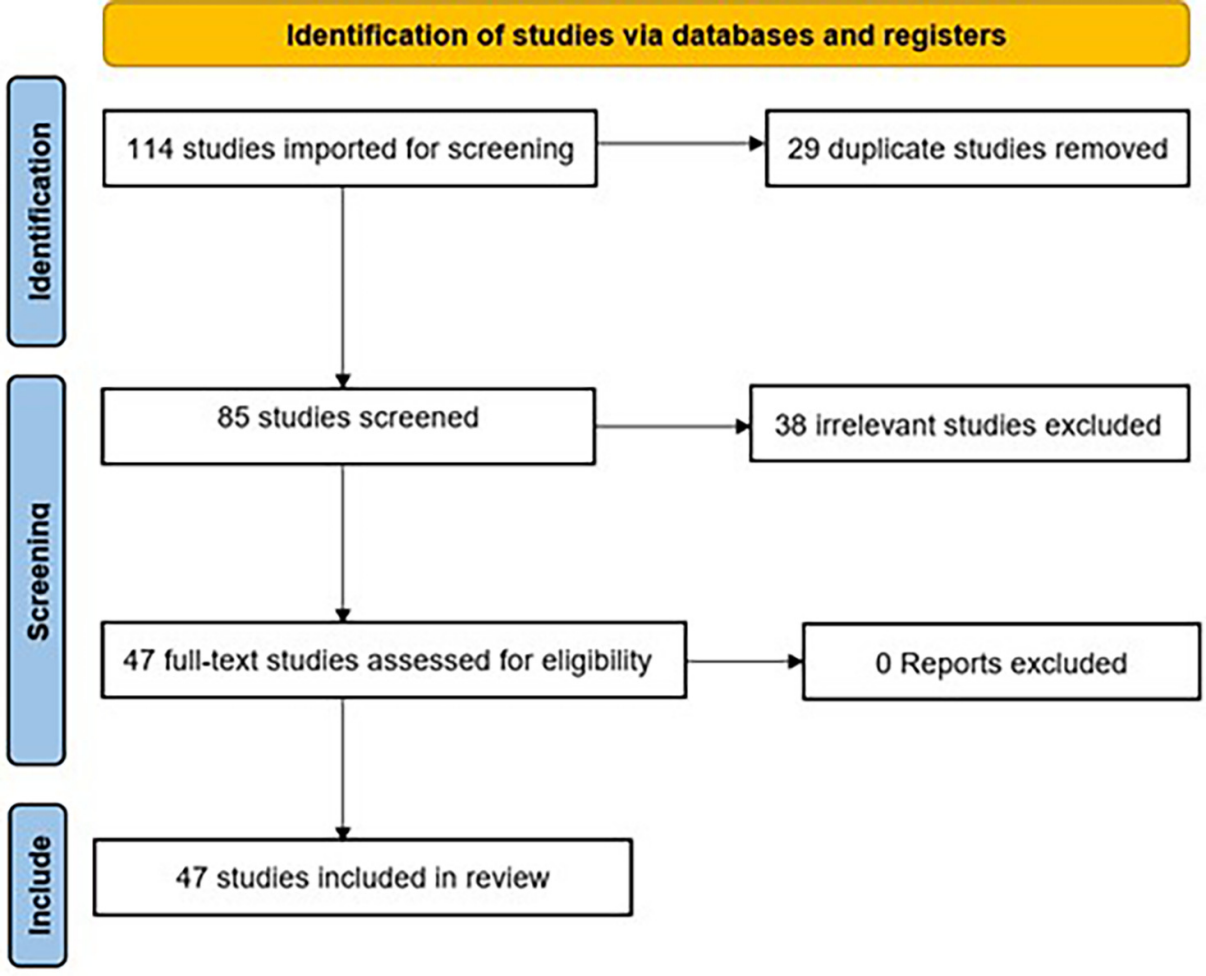
PRISMA flow diagram.

A systematic screening process was conducted following PRISMA guidelines. A total of 114 studies were initially identified by the search strategy. After removing 29 duplicate studies, 85 unique studies remained for further evaluation. During the screening phase, 38 studies were excluded due to irrelevance, leaving 47 full-text articles for eligibility assessment. All 47 studies met the inclusion criteria, and none were excluded at this stage. Consequently, 47 studies were included in the final review, as shown in Figure 1 and Table 1.

**Table 1:** Summary of articles reviewed.

No.	Author	Year of Publication	Topic	Study design	Study Population	Male	Female
1	Adeyemi et al.	2011	Disease risk/resistance	Randomised controlled trial	1,358	731	627
2	Adinma et al.	2015	Disease risk/resistance	Cohort study	315	187	128
3	Akinbami et al.	2012	Disease risk/resistance	Cohort study	4,042	1,535	2,507
4	Alex et al.	2019	Patterns of AM use	Cross sectional study	184	115	69
5	Alhaji et al.	2019	AMS behavior	Cross sectional study	384	328	56
6	Alobu et al.	2014	Health seeking behaviour	Case control study	1,668 985	602	383
7	Aniekwu et al.	2002	Health seeking behaviour		Not indicated	Not indicated	Not indicated
8	Antai et al.	2012	Health seeking behaviour	Cross sectional study	33,385		33,385
9	Anucha et al.	2021	Disease risk/resistance	Randomised controlled trial	236	95	141
10	Anugwom et al.	2016	Health seeking behaviour	Qualitative research	Not indicated	Not indicated	Not indicated
11	Aworh et al.	2021	Disease risk/resistance	Cross sectional study	144	104	40
12	Azuh et al.	2015	Health seeking behaviour	Randomised controlled trial	260	0	260
13	Daniel et al.	2005	Health seeking behaviour	Cohort study	103 Can’t view		
14	Duru et al.	2016	Disease risk/resistance	Cohort study	1,025	549	476
15	Ebong et al.	2021	Health seeking behaviour	Cross sectional study	5,650	653	4,997
16	Eteng et al.	2022	Health seeking behaviour	Text and opinion	Not indicated	Not indicated	Not indicated
17	Fatiregun et al.	2009	Health seeking behaviour	Cohort study	1,254	690	564
18	Fatiregun et al.	2010	Health seeking behaviour	Cross sectional study	102	41	61
19	Idowu et al.	2011	Patterns of AM use		60	23	37
20	Ifebunandu et al.	2012	Health seeking behaviour	Cohort study	671	370	301
21	Jombo et al.	2011	Disease risk/resistance	Cohort study	565	264	301
22	Jumbo et al.	2013	Healthcare Access/Use	Cohort study	2,625	1,612	1,013
23	Mmari et al.	2010	Health seeking behaviour	Cohort study	538	162	376
24	NosayabaOsazuwa- Peters et al.	2012	Health seeking behaviour	Cross sectional study	90	49	41
25	Nwankwo et al.	2019	Health seeking behaviour	Randomised controlled trial	1,116	Can’t view	Can’t view
26	Ogbonda et al.	2020	Health seeking behaviour	Cross sectional study	391	204	187
27	Ogundari et al.	2014	Health seeking behaviour	Randomised controlled trial	18,883	Not indicated	Not indicated
28	Okonkwo et al.	2010	Patterns of AM access	Non-randomised experimental study	23	15	8
29	Okonofua et al.	2018	Health seeking behaviour	Cross sectional study	1,408		1,408
30	Okoronkwo et al.	2014	Healthcare Access/Use	Cross sectional study	360	101	259
31	Oladeinde et al.	2011	Disease risk/resistance	Randomised controlled trial	514	49	465
32	Onah et al.	2014	Healthcare Access/Use	Cross sectional study	411	251	160
33	Onah et al.	2018	Healthcare Access/Use	Cross sectional study	411 households	Not indicated	Not indicated
34	Onanuga et al.	2012	Disease risk/resistance	Randomised controlled trial	137 urine samples	Not indicated	Not indicated
35	Onwuchuluba et al.	2022	Health seeking behaviour	Cross sectional study	48	20	28
36	Orisaremi et al.	2016	Healthcare Access/Use	Other: Purposive Sampling	40	21	19
37	Orpin et al.	2019	Healthcare Access/Use	Qualitative research	16	6	10
38	Oshi et al.	2015	Healthcare Access/Use	Cohort study	1,668	963	705
39	Oshi et al.	2016	Health seeking behaviour	Non-randomised experimental study	56	10	46
40	Peters et al.	2002	Health seeking behaviour	Cohort study	2,309	1,527	782
41	ReAct et al.	2020	Disease risk/resistance	Case control study	Not indicated	Not indicated	Not indicated
42	Tula et al.	2014	Disease risk/resistance	Randomised controlled trial	101	46	55
43	Ukwaja et al.	2013	Health seeking behaviour	Cross sectional study	450	246	204
44	Umar et al.	2012	Health seeking behaviour	Cross sectional study	242	132	110
45	Varkevisser et al.	2009	Disease risk/resistance	Cohort study	Not indicated		
46	Wang et al.	2022	AMS behavior	Cross sectional study	211	Not indicated	Not indicated
47	Yaya et al.	2019	Healthcare Access/Use	Qualitative research	179	104	75

Search strategy key words:

1. Antimicrobial Resistance
2. Antibiotic resistance/
3. Antibiotic prescription/
4. Antimicrobial Stewardship/
5. Women/
6. Gender/
7. Sex/
8. Female/
9. Male/
10. Treatment seeking behavior/
11. Health$ access/
12. Antimicrobial access/
13. Antibiotic access/
14. Urinary tract infection
15. Tuberculosis/
16. Sexually Transmitted infections
17. Infectious disease treatment

## Results

Data was extracted on article characteristics, area of AMR focus and results of gender. Studies were then grouped by condition/infectious disease under study, area of AMR, and study design, along with the measures used and broad findings. We did not identify any previous reviews (systematic or otherwise) of this topic in the literature.

After duplicates were removed, a total of 85 citations were identified from searches of electronic databases. Based on the title and the abstract, 38 were excluded as irrelevant, with 47 full text articles to be retrieved and assessed for eligibility. Upon full text review, all 47 were found to be eligible and no exclusions were made.

Of these 47 studies, 19 had an explicit gender approach in their design whereas 27 merely included gender as a variable. The majority were cross-sectional studies (16), followed by cohort studies (12) and Randomised (8) and non-randomised control trials (2). The frequency of publications increased over time as shown in Table 2. Mycobacterial infections (tuberculosis, leprosy) were well represented, as well as sexually transmitted infections (including HIV) and urinary tract infections.

**Table 2:** Distribution of article types covering gender in AMR.

Study design	Case control study	Qualitative research	Cross sectional study	Cohort study	Randomized controlled trial	Non-randomized experimental study
	2	3	17	12	8	2
Topic	AMS behaviour	Disease risk/resistance	Health seeking behaviour	Healthcare Access/Use	Patterns of AM use	
	4	11	20	10	2	
Publication	Number	7	10	20	7	2
	Year	2021–222	2016–2020	2011–2015	2006–2010	2000–2005

### Gender differences in risk of infection and AMR

There are gender differences in risk of infection and AMR for different infections. Women have a 7 times higher risk of UTI for biological/anatomical reasons, accounting for 60% of UTI infections. Women also experience more severe antimicrobial resistance, with 30% vancomycin-resistant *Staph aureaus* in women with UTI, compared to just 8% in men (26, 27). On the other hand, men are more likely to be infected with TB and less likely to comply with treatment guidelines for both TB and leprosy (28–30). The major risk factors for infection also differ by sex—HIV co-infection for women and urban residence for men (41).

Nigeria has one of the highest maternal mortality rates in the world, accounting for 19% of global maternal deaths. Yet overwhelmingly, men retain the power to make decisions about contraceptive use which are implemented by women (22, 31). Women's experiences associated with pregnancy, abortion and childbirth may put them at increased risk of antibiotic resistance. These include harmful practices by health care workers that can result in infection for example Oduenyi et al. (31) report >30% women experienced unnecessary routine episiotomies, manual exploration or lavage of the uterus during childbirth.

Inherent gender discrimination in health service delivery further affects women's health seeking behaviour through sub-standard levels of care: 65% of women reported physical and/or verbal abuse from HCWs at facilities (31). This expectation of poor treatment by HCWs is the reason 11%–20% women choose not to attend health facilities when ill (20, 32). Workplace violence and gender discrimination experienced by healthcare workers themselves serves to normalize and reinforce these behaviours—8% report violent incidents including rape, sexual assault and beatings (31). Women comprise the majority of the healthcare workforce but the majority of supervisors are male (31, 33).

### Gender inequality

Gender inequality limits women's access, choice, agency and autonomy in accessing healthcare services. Low agency in decision making and financial dependence on male partners results in reduced attendance at health facilities for women, lower immunization rates in children and unmet needs for contraception in Nigeria (22, 31, 34, 35). 15%–36% of women did not attend health facilities when ill or pregnant because they did not have permission from their spouse (20, 32). Female-headed households spent a higher proportion of their income on healthcare (and education) than male-headed households, but also reported 2.5 times higher untreated morbidity due to cost, compared to male-headed households.

Patterns of health seeking behaviour also differ by gender in different settings across the country: women were more likely than men to seek treatment from formal healthcare facilities where they mostly provide maternal & child health services and are perceived as being “for women” (32, 34). In other settings, men were twice as likely to seek healthcare when ill and 20 times more likely to use the formal sector than women due to their sole control of household resources; women were more likely to patronize PPMVs (21, 36).

Despite comparable levels of knowledge on HIV & STIs, men were twice as likely to seek treatment compared to women due to higher stigmatization and fear of spouses' finding out (40% young women in the study were married compared to just 17% of young men) (34). This is reflected in a 2011 study of people self-reporting STIs where 86% of men based their report on a medical diagnosis compared to just 6% of women. Women were also less likely to inform their partners of their status (37).

## Discussion

Results show clear gender differences in all aspects of AMR in Nigeria, including the burden of AMR, the behavioural patterns that promote it, and the lived experiences of healthcare workers. These are clearly well-recognized patterns, yet such recognition often occurs without a corresponding analysis of how gender might reinforce these vulnerabilities (38). In Nigeria, where 98% of HCWs believe men should be involved in family planning (and 60% believe women cannot take these decisions without their spouse) only 10% encourage women to bring their partners along to consultations (31).

Studies and interventions on AMR rarely show these gendered nuances, despite the strong evidence for gender as a social determinant of health. More attention is required to how gender perspectives in awareness of AMR, antimicrobial consumption and stewardship, health care, livestock production affect our ability to reduce and prevent antimicrobial resistance. More attention is also required to increase the number of women working on AMR and STEM in general and improve the supporting enabling environment for them to thrive.

Nigeria has quite significant representation of women and women-led initiatives working in AMR research and control at all levels, across the public, private and non-profit sectors. Expert consultations among this target group investigate the gendered aspects of women working in STEM, AMR research and control, specific challenges. They also offer recommendations to better understand gender aspects of AMR in Nigeria and leverage on them to accelerate the fight against this priority health threat.

There are both positive and negative aspects of being a female STEM professional working in AMR. In line with the workplace norms in Nigeria (39), tokenism, being overlooked or taken less seriously than male colleagues, being subjected to inappropriate comments by male colleagues and then made to feel this is culturally acceptable were commonly cited instances, both in professional life, and during education and STEM training.

On the other hand, supporting and being supported by other women in the workplace and mentorship by accomplished female scientists locally and globally, have been very positive.

It has been clearly demonstrated that having a close network of supportive women is key to career success for women (40). This is particularly important to encourage continued participation of women in in STEM careers and AMR. The One Health approach and multisectoral nature of the AMR space in Nigeria has also provided beneficial opportunities to build bridges and share experiences with colleagues in other sectors (including other women).

On the other hand, some of the barriers and negative experiences mean that women must still work harder to prove their value in the fight against AMR.

The following are recommended to address the low consideration of gender and inclusivity in AMR in Nigeria:

### Equality, diversity and inclusion in AMR behavioural change

Awareness and behavioural change communication is key in the fight against AMR. To make this more effective, messaging should be tailored to different groups within the general public audience, including men, women and youths. Efforts should be made to increase women and youth inclusion opportunities in all areas of behavioural change, including innovation, research and development. Messaging also needs to be localised and evidence-based, delivering, context—specific messages and scenarios in local languages, which take gender differences into account.

There is a need to identify diverse AMR champions to give this often silent, abstract problem a human face by sharing their personal stories to encourage behavioural change. AMR can be difficult to conceptualise hence the need to be able to connect to the stories of others to appreciate the looming dangers.

### Governance

Comprehensive mapping of projects, programmes, expertise and resources for AMR is necessary to clearly determine levels of gender sensitivity and plan for improvement of same. Increased political commitment is also important to guarantee implementation the National Action Plan on AMR at subnational and community levels where gender considerations are most significant. Finally, inclusion of gender considerations in design, implementation, evaluation and reporting of AMR interventions must be improved for gender mainstreaming.

### Inclusivity in AMR prevention and control

To improve inclusivity in AMR interventions, gender-sensitive stakeholder mapping is very useful, to strengthen the ecosystem of women in STEM & AMR. Women in this field need more opportunities to connect and support each other. Mentorship and networking amongst women should be promoted both nationally and globally. In terms of impact, existing programs and organizations should be supported to reach more women, especially in areas with significant barriers to girls' educations and women's workforce participation. Investments in gender equality in access to and experiences of STEM education from the basic educational levels (primary and secondary) to advance level promote participation of girls. All relevant institutions and workplaces should establish and implement equality, diversity and equity policies to improve workplace experiences for women. This will lead to increased participation of qualified women can be employed and supported to remain productive at their at all stages of their lives.

## Conclusion

There is clear but limited evidence of the gender differences within the root causes and burden of AMR in Nigeria, as well as within the workforce responsible for preventing and controlling it. Allocating resources to improve the inclusion of gender and inclusivity in the study and practice of AMR prevention and control is key. Together with the recommendations proffered here, this will produce much needed evidence and guidance to more effectively combat this priority health problem.

## Data Availability

The original contributions presented in the study are included in the article/Supplementary Material, further inquiries can be directed to the corresponding author.
